# Tuna sidestream valorization: a circular blue bioeconomy approach

**DOI:** 10.1007/s11356-023-28610-w

**Published:** 2023-07-11

**Authors:** Abhilash Sasidharan, Turid Rustad, Giovanni M. Cusimano

**Affiliations:** 1 Department of Biotechnology and Food Science, Norwegian University of Science and Technology (NTNU), 7491 Trondheim, Norway; 2 Department of Fish Processing Technology, KUFOS, Kerala, India; 3 AquaBioTech Group, Mosta, MST 1761 Malta

**Keywords:** Seafood sustainability, Tuna processing, Animal nutrition, Bioactive peptides, Collagen, Protein recovery

## Abstract

Tuna is an economically significant seafood, harvested throughout the world, and is heavily traded due to its high nutritional quality and consumer acceptance. Tuna meat is rich in essential nutrients such as amino acids, polyunsaturated fatty acids (PUFA), and trace minerals. The huge volume of solid and liquid sidestreams generated during the processing stages of tuna is creating environmental and socioeconomic challenges in coastal areas. Different products such as fish meal, protein hydrolysates, collagen, enzymes, oil, and bone powder can be produced from tuna sidestreams. Using different nutrient recovery technologies like enzymatic hydrolysis, chemical processing, and green technologies, various categories of product value chains can be created in line with the conventional processing industry. This review attempts to provide a route map for the tuna industry for achieving the circular blue-bioeconomic objectives and reorient the irregular utilization pattern into a sustainable and inclusive path.

## Introduction

Seafood is an essential component of most food security programs being a comparatively cost-effective protein resource worldwide. In 2020, the total world seafood production of 179.8 million metric tons (MT) contributed around 157.4 MT directly for human consumption (at 20.2 kg per capita rate) (FAO 2022). The European Commission (2018) predicts that by 2050, the demand for seafood may increase by up to 60% along with the increasing global population, which is expected to reach 9.8 billion by that time. Seafood supply is confronting numerous challenges such as population-induced demand pressure, changing consumer preferences, overfishing, bycatch, species depletion, aquatic pollution, global warming, biodiversity alterations, and acidification of ocean water (Venugopal 2022). The key issue often associated with aquatic food production is the production of large amount of sidestreams which creates a huge loss of nutrients if discarded irresponsibly. The sidestreams are the unutilized parts like the skin, bones, and viscera which are equally rich in nutrients as the edible parts but are frequently discarded (Venugopal and Sasidharan 2022). Systematic attempts to transform the existing aquatic food production systems into a sustainable mode are therefore required (FAO et al. 2021). In this scenario, new technological approaches which at the same time reduce food loss and wastage and also can valorize the resulting sidestreams into valuable products should be introduced (Venugopal 2022).

The tuna, occasionally known as the “chicken of the sea,” is one of the major marine fish varieties given its contribution toward the seafood trade in value, volume, and nutritional significance. It belongs to the Scombridae family and consists of around 15 species, the major ones being the albacore tuna (*Thunnus alalunga*), bigeye tuna (*Thunnus obesus*), Atlantic bluefin tuna (*Thunnus thynnus*), Pacific bluefin tuna (*Thunnus orientalis*), skipjack tuna (*Katsuwonus pelamis*), and yellowfin tuna (*Thunnus albacares*). These tuna species are distributed widely along Pacific, Atlantic, and Indian Oceans (Allain et al. 2016). The major harvesting methodologies involve purse seines, gillnets, long lines, hand lines, and pole lines. FAO reported tuna catches of 7.8 million tonnes in 2020 (FAO 2022). In 2020, the worldwide export of tuna and tuna-like fishes (Bonitos and Bill fishes) was worth USD 14.6 billion contributing around 9.7 percent by value of total aquatic product exports (FAO 2022).

The market trade of the species is divided into two broad categories, processed and preserved tuna meat and the superior quality fresh tuna meat, meant for the sushi and sashimi market. The processed and preserved category consists of canned, fresh, or frozen, largely based on loins which constitute up to 50% of the fish (Herpandi et al. 2011). Atlantic, Pacific, and Southern bluefin tuna are preferred for the sushi and sashimi market and are therefore the most valuable (Metian et al. 2014). The higher demand and reduced catches over the years have resulted in an increasing trend in aquaculture of bluefin tuna especially in Japan, Spain, Malta, Croatia, Mexico, and Australia (FAO 2022).

The global tuna industry is facing numerous sustainability challenges. As the tuna are highly migratory and predatory, any sustainability issue associated with the stocks could percolate down to the entire pelagic ecosystem of the oceans. Tuna fishing vessels are usually industrial trawlers with significant ecological impact associated with their bycatch, and in addition, around 43% of tuna fish stocks are exploited at unsustainable levels (FAO 2020). For the fisheries to be operated at a sustainable level, it is important with optimal utilization of the available tuna resources. Environmental, social, and economic sustainability measures must be introduced in the consumption pattern. The recovery of valuable nutritional components from the otherwise discarded sidestreams through different valorization methods and reintroducing the components into human food chain could address some of the nutrition deficiency issues of many food-insecure populations (Hicks et al. 2019). Other than the handling cost involved, food loss and sidestream generation are generally attributed to insufficient logistics, technological inadequacy, and consumer behavior (Chauhan et al. 2021). These parameters can also vary geographically, for example, insufficient logistics and technological inadequacy are generally attributed to underdeveloped or developing countries and poor consumer awareness with the developed nations (Gustavsson et al. 2011).

In the underdeveloped and developing countries, the most significantly affected resources are the water bodies adjacent to the processing facilities as the overloading of the nutrients from seafood sidestreams discarded may result in eutrophication and increase in water pollution indicators (Sasidharan et al. 2013). In the case of European nations, the newly introduced measures like landing obligation regulations prevent open water discards (EC 2021), and the landed discards and sidestreams create new challenges regarding handling and processing. The economic perspective of food loss and wastage (FLW) should also be considered while addressing the management of sidestreams. The blue economic approach involves ocean resource utilization patterns targeted for attaining economic growth, enhanced employment opportunities, ocean ecosystem health, and interlinking of all the traditional and evolving engagements including fisheries associated with it (World Bank 2017). Fisheries, aquaculture, and associated processing systems form an integral part of the blue economy. They have interlinking influence with other components along with the externalities that are generated. One of the ten social injustices as described by Bennett et al. (2019) is the challenge of pollution and sidestreams, which has the potential to upset the blue economy balance. Even though there are many works which discuss seafood sidestream management in general (Thirukumaran et al. 2022; Sasidharan and Venugopal 2020) and raw material specifically (Zou et al. 2023), a comprehensive review on the valorization potential of tuna sidestreams is not available. This could be of interest for the academic and industrial community involved in tuna-related fisheries, aquaculture, and processing. In light of this, this article attempts to evaluate the variety of sidestreams generated during the different tuna pre-processing, processing, and preservation methods and explore the technologies available to recover the nutritional constituents effectively and thus to provide the stakeholders to frame productive resource recovery road maps for sustainable utilization of the tuna fish resources.

## Yield and recovery options for tuna sidestreams from processing operations

**Fig. 1:**
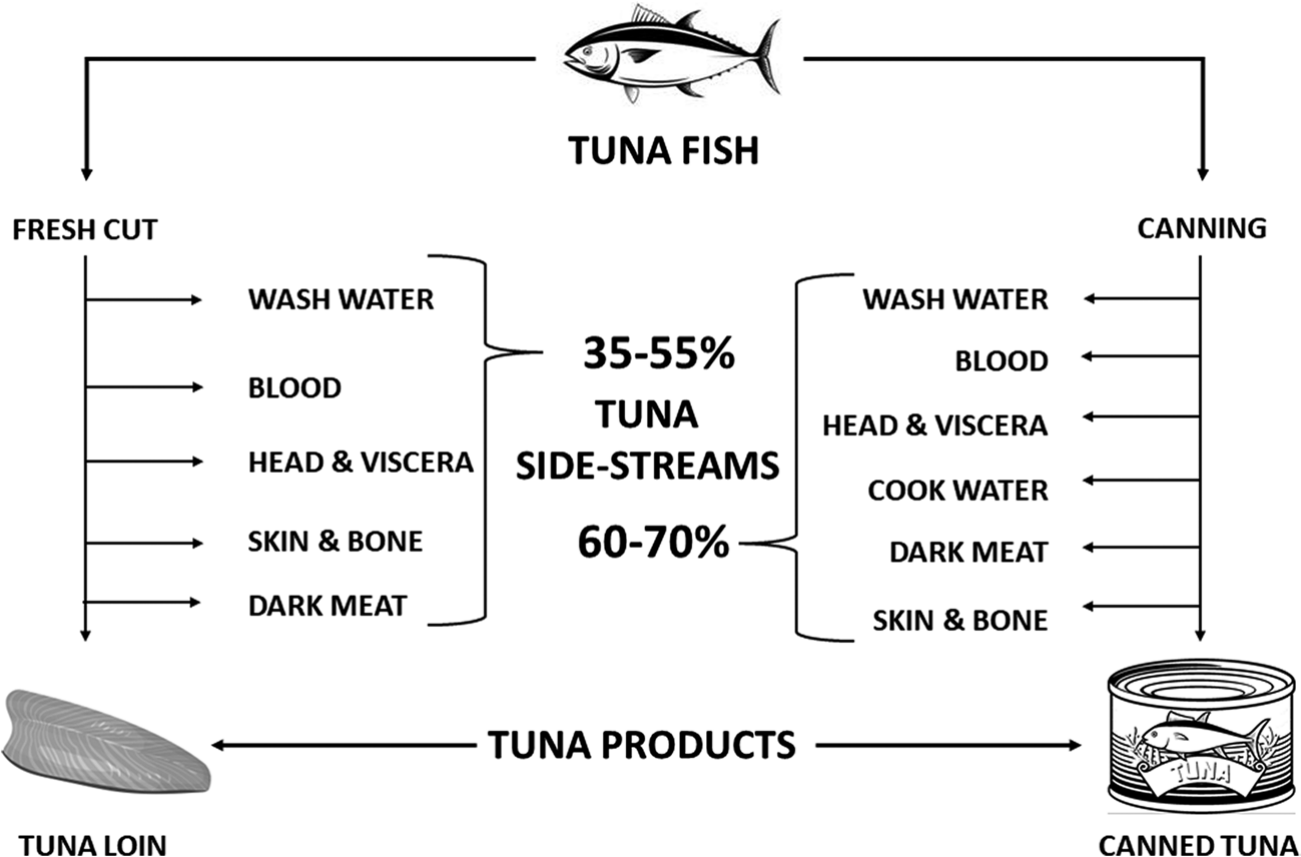
Tuna sidestream generation and yield

Different processing and pre-processing methods yield different quantities of tuna sidestreams. The fillet yield of yellowfin tuna (*Thunnus albacares*) is estimated as 45% and the rest (55%) consist of belly flap, bone, skin, tail, gut, and head (Murthy et al. 2014). Canning, one of the important value addition operations in tuna processing, is reported to produce up to 70% of solid sidestream comprised of dark meat, head, skin, and bone (Sasidharan and Venugopal 2020). Sayana and Sirajudheen (2017) observed that approximately 36% of sidestream is generated during tuna processing with head being 17%, skin 8%, viscera 5%, bones 4%, and fins 2%. Fluence (2019) reported that a tuna cannery processing around 200 tons of raw material per day could discharge around 1300 m^3^ effluents. Figure 1 represents the flow of tuna sidestreams during various processing activities. Tuna is nutritionally rich in all the essential nutritional components. Table 1 depicts the proximate composition of various commercial tuna species, and it is evident that the tuna fish is a good source of high-value protein. The amino acid and fatty acid profiles of different commercial tuna species (Tables 2 and 3) confirm the presence of essential amino acids as well as fatty acids such as eicosapentaenoic acid (EPA) and docosahexaenoic acid (DHA). This shows that the sidestreams generated from the tuna processing operations could be considered equally valuable in nutritional components as the edible portions. The hydrolysates prepared from tuna processing sidestream possess good nutritional attributes (Herpandi et al. 2011) with considerable proportion of essential fatty acids with yield levels up to 7% (Taati et al. 2018). The dark muscle of tuna is one of the important solid sidestreams generated from the processing operations. Tuna dark muscle has been reported to make up to 48% of the body weight in species like yellowfin tuna. The distinct biochemical difference regarding the tuna dark muscle when compared to light muscle is the high fat, blood pigment, and iron content, which makes the tuna dark muscle a potential product for extracting value-added components like PUFA (Messina et al. 2022).

**Table 1:** Proximate composition of commercial tuna species

Sl. no.	Tuna species	Moisture	Protein	Lipid	Ash	Reference
1.	Yellowfin tuna (*Thunnus albacares*)	73.6	23.5	1.9	1.5	Peng et al. 2013
2.	Bigeye tuna (*Thunnus obesus*)	72.9	23.7	2.1	1.8	Peng et al. 2013
3.	Atlantic bluefin tuna (*Thunnus thynnus*)	63.3	21	11	4.7	Topic Popovic et al. 2012
4.	Albacore tuna (*Thunnus alalunga*)	58.8	23.5	16	1.6	Wheeler (2002)
5.	Pacific bluefin tuna (*Thunnus orientalis*)	68.6 ±	25	0.6	1.9	Roy et al. 2010
6.	Skipjack tuna (*Katsuwonus pelamis*)	73.3	24	0.4	1.4	Mahaliyana et al. 2015

**Table 2:** Amino acid composition of different body parts of commercial tuna species

Amino acids	Yellowfin tuna (*Thunnus albacares*)	Bigeye tuna (*Thunnus obesus*)	Atlantic bluefin tuna (*Thunnus thynnus*)	Pacific bluefin tuna (*Thunnus orientalis*)	Skipjack tuna (*Katsuwonus pelamis*)
Serine	3.23	3.12	3.72	26.7	2.69
Tyrosine	3.19	3.14	1.95	19.8	2.54
Proline	3.08	2.99	5.19	2.7	–
Aspartic acid	8.29	8.11	12.45	5.2	7.35
Glutamic acid	12.45	11.28	10.90	73.7	11.22
Glycine	3.75	3.66	13.96	3.2	4.83
Alanine	5.14	5.02	22	32.3	5.04
Cystine	0.44	0.49	–	–	–
Arginine	5.11	4.96	1.98	34.2	4.85
Threonine	3.85	3.75	6.85	24.8	3.30
Valine	4.54	4.63	8.2	19.1	4.25
Methionine	2.55	2.76	2.04	15.4	2.16
Isoleucine	4.06	4.01	9.15	15.5	3.89
Leucine	6.99	6.86	17.75	38.7	5.89
Phenylalanine	3.30	3.32	5.88	21.1	3.23
Lysine	8.19	7.93	14.86	41.3	6.29
Tryptophan	0.88	0.86	–	7.0	–
Histidine	5.49	5.26	6.10	17.4	6.72
References	Peng et al. 2013	Peng et al. 2013	Balestrieri et al. 1978	Cho et al. 2022	Doe et al. 2020

**Table 3:** Fatty acid composition of commercial tuna species

Fatty acids	Yellowfin tuna (*Thunnus albacares*)	Bigeye tuna (*Thunnus obesus*)	Atlantic bluefin tuna (*Thunnus thynnus*)	Pacific bluefin tuna (*Thunnus orientalis*)	Skipjack tuna (*Katsuwonus pelamis*)
14:0	1.3	1.66	2.92	2.9	2.02
15:0	0.6	0.73	–	0.4	0.66
16:0	26.18	24.55	16.21	17.8	21.88
16:1	3.08	3.42	3.98	3.7	2.49
16:2	–	–	0.58	–	–
16:4	–	–	0.68	–	–
17:0	1.47	1.07	–	–	–
17:1	0.83	0.92	–	0.4	–
18:0	–	–	4.95	6.2	11.69
18:1	20.32	24.19	25.85	15.5	10.03
18:2n-6	1.31	0.92	1.25	1.0	1.38
18:3n-3	–	–	0.86	1.2	0.13
18:4n-3	–	–	1.54	–	–
20:0	0.55	0.85	–	6.3	–
20:1n-7	–	–	4.29	–	–
20:2	–	–	–	–	–
20:4n-6	–	–	1.05	1.3	0.32
20:4n-3	–	–	0.87	–	
20:5n-3	1.25	3.27	6.51	6.9	4.74
22:1n-7	–	–	3.51	–	–
22:5n-6	–	–	1.74	–	–
22:6n-3	16.91	20.22	16.24	22.3	35.66
References	Peng et al. 2013	Peng et al. 2013	Topic Popovic et al. 2012	Nakamura et al. 2007	Mahaliyana et al. 2015

### Protein recovery options

The technique to recover the protein from any seafood solid sidestream is to either convert it into a meal with oil as one of the products or subject it to partial or complete hydrolysis with or without oil recovery. The conventional meal preparation involves vigorous high-temperature cooking processes resulting in the loss of valuable nutrients (Abraha et al. 2018). Protein hydrolysis by using endogenous or commercial enzymes could be utilized to produce valuable protein-based ingredients from the tuna sidestreams. Even though autolysis-assisted hydrolysis has been frequently used, enzymatic hydrolysis with exogenous enzymes has evolved as the common and commercially applied technology for the recovery of essential and biologically active protein components (Chalamaiah et al. 2012).

#### Tuna silage

The nutritional potential of tuna silage has been demonstrated in many studies. Spanopoulos-Hernandez et al. (2010) prepared biological silage using smoked yellowfin tuna filleting residue fermented with sugar cane molasses and *Lactobacillus casei* strain Shirota as the commercial inoculum for six days, which produced a silage with acceptable properties for application in animal feed. Ramli (2014) was successful in preparing fermented silage from tuna sidestreams using inoculums of *Lactobacillus plantarum* (1A-2) and *Lactobacillus plantarum* (1BL-2) in different culture concentrations, resulting in nutritionally superior hydrolysate. Mousavi et al. (2013) prepared tuna silage from canning sidestreams of three different species of tuna using three different methods and found that the biological method was the optimum with ideal pH (4) suitable for shrimp feed applications. Filipe et al. (2017) prepared acid silage from tuna viscera achieving 61.7% solubilization and 88.5% digestibility of the crude protein.

#### Tuna hydrolysate

The process of hydrolysis cleaves complex protein molecules down to peptide and amino acid components which could be commercially utilized to develop food and feed ingredients for human as well as animal populations. Saidi and Ben Amar (2016) utilized enzymatic hydrolysis with Prolyve BS enzyme to transform the tuna dark muscle sidestream into tuna protein hydrolysate (TPH). The hydrolysate exhibited elevated oil and water-binding capacity, emulsifying capacity, foam stability, higher radical scavenging activity, and a higher iron chelating activity in comparison with other similar fractions. A TPH was prepared from the dark muscle of tuna by Saidi et al. (2013) using Alcalase and Neutrase as the enzyme producing a TPH with a significant amount of a peptide fraction of about 1–4 kDa of molecular weight with a balanced composition of essential amino acids (EAA). Chi et al. (2015) observed that the peptide fraction with a molecular weight less than 1 kDa in the tuna dark muscle hydrolysate prepared using the Neutrase enzyme exhibited superior radical scavenging activity. Chotikachinda et al. (2018) prepared tuna viscera hydrolysate for application in aquatic feed using different endo and exopeptidase enzyme sources with different degrees of hydrolysis from *Bacillus licheniformis*, *Aspergillus oryzae*, and *B. amyloliquefaciens*, with positive nutritional effects. Table 4 indicates the key highlights regarding the techniques adapted for tuna sidestream hydrolysate recovery.

**Table 4:** Technique adaptation for tuna sidestream protein hydrolysis

Sl. no.	Sidestream source	Enzyme/substrate/technique	Properties/results	References
1	Tuna dark muscle	Prolyve BS enzyme in combination with ultrafiltration (UF) and nanofiltration (NF) (10 min @ 50°C)	Oil and water binding capacity, emulsifying capacity, foam stability, radical scavenging activity, iron chelating activity	Saidi and Amar 2016
2	Tuna dark muscle	Alcalase and Neutrase (1 h @ 55°C)	Peptide fraction with a molecular weight of 1–4 kDa and balanced composition of essential amino acids (EAA)	Saidi et al. 2013
3	Tuna viscera	Autolysis (10 days @ 33°C, 35°C, and 55°C)	Hydrolysate with radical scavenging activity	Detkamhaeng et al. 2016
4	Tuna dark and white muscle	Papain (240 min @ 55°C)	Hydrolysate with high protein content (72%)	Dana et al. 2019
5	Tuna viscera	Endoproteinase (source: *Bacillus licheniformis*), endoproteinase and exopeptidase (source: *Aspergillus oryzae*), and endoproteinase (source: *B. amyloliquefaciens*) (10 min @ 50°C)	Rich in essential nutrients for feed application	Chotikachinda et al. 2018

### Application of recovered tuna protein hydrolysate in animal nutrition

Tuna sidestream meal and hydrolysates have been widely used as a nutritional component in different animal dietary formulations. Even though different nutritional studies incorporating tuna sidestream as a protein supplement has been reported in various animal species, the studies were mainly concentrated on pig and poultry nutrition (Anuraj et al. 2014; Widjastuti et al. 2011) and feed for popular aquaculture species such as Pacific white shrimp (*Litopenaeus vannamei*) (Hernández et al. 2017), Asian seabass or barramundi (*Lates calcarifer*) (Chaklader et al. 2021), and Nile tilapia (*Oreochromis niloticus*) (Kim et al. 2019). The aquatic feed industry provides a commercially promising arena for tuna sidestream valorization as a potential fish meal substitute.

#### Pig and poultry nutrition

Anuraj et al. (2014) studied the impact of dietary inclusion of tuna sidestream silage on the growth parameters of Large White Yorkshire pigs and found tuna silage to be an excellent source of nutrition for pigs. In a feeding experiment with thirty-six weaned Large White Yorkshire piglets, tuna sidestream silage mixed with rice bran was used which generated comparable growth parameters with that of standard fish meal-based diet (Yathavamoorthi et al. 2015). Widjastuti et al. (2011) utilized tuna sidestream silage prepared from *Thunnus atlanticus* as a dietary ingredient for broiler chicken in varying concentrations concluding that 4% inclusion of tuna sidestream silage gave positive response in broilers regarding body weight and meat protein conversion.

#### Shrimp nutrition

Fish meal replacement studies were conducted by Hernández et al. (2017) by utilizing tuna sidestream silage and soybean meal in the diet of Pacific white shrimp (*Litopenaeus vannamei*). The 41-day feed trial inferred that 25% inclusion of tuna sidestream silage along with 75% of soybean meal generated comparable growth results with that of fish meal control. Lactic acid fermentation methodology was used to prepare tuna sidestream hydrolysate, and this was further used as a feed ingredient in the diet of Pacific white shrimp (*Litopenaeus vannamei*) in combination with porcine meat meal (Hernández et al. 2011). It was observed that the *L. vannamei* shrimps fed with feed supplemented with 5% of tuna sidestream hydrolysate reported a significant increase in weight gain (8.6 g), feed conversion ratio (FCR) (1.3), and specific growth rate (5.1% per day) compared to the control. These results were credited to the effect of tuna sidestream hydrolysate which served as an attractant as well as an enhancer of protein digestibility and protein quality of the formulations.

#### Fin fish nutrition

Six-week feeding trials were conducted by Chaklader et al. (2021) to understand the fish meal replacement potential of poultry sidestream meal supplemented with tuna sidestream hydrolysate and insect larvae, on the growth, nutritional, sensory, and gut microbial characteristics of juvenile barramundi (*Lates calcarifer*). The study demonstrated that simultaneous combination of tuna sidestream hydrolysate and insect larvae could significantly improve the effect of poultry sidestream meal-based diets on the growth, meat quality, gut health, and immune traits of barramundi juveniles in comparison to fish meal. Siddik et al. (2019) used tuna sidestream hydrolysate as a co-supplement with poultry sidestream meal in the diet of *Lates calcarifer* and found that the poultry and tuna sidestream meals when complemented together generated significant increase in growth, immunity, intestinal health, and *Vibrio harveyi* resistance in juvenile barramundi. An eight-week feeding trial was conducted by Tola et al. (2022) to examine the effect of tuna hydrolysate supplementation on growth performance and health status of juvenile barramundi (*Lates calcarifer*). The study demonstrated that a 2.5% tuna hydrolysate dietary supplementation is sufficient to enhance the diet palatability of a low fish meal diet formulated with 55% replacement of fish meal with soybean meal without negative impact on feed intake and growth performance of juvenile barramundi. Kim et al. (2019) demonstrated that tuna sidestream-based ingredients are a potential candidate for fish meal replacement in tilapia (*Oreochromis niloticus*) aquaculture as the heavy metal residues are within the food safety limits (<0.5 mg kg^−1^) which is estimated to significantly reduce the feed expenditure along with other fish meal alternatives in tilapia farming. Mokolensang et al. (2021) conducted feeding trials on juvenile tilapia with a combination of tuna sidestream meal and blood meal. The study revealed that the combination of 20% of each significantly increased the weight gain and specific growth ratio. Table 5 gives an overview of tuna sidestream products application in animal nutrition.

**Table 5:** Livestock feed applications of tuna sidestreams

Sl. no.	Sidestream	Sidestream by-product	Livestock category	Highlights of the study	References
	Tuna sidestream solids	Tuna sidestream acid silage	Large White Yorkshire pigs	Dried tuna silage reported identical results with dried fish in terms of growth rate and FCR.	Anuraj et al. 2014
	Tuna sidestream solids	Tuna sidestream acid silage	Large White Yorkshire pigs	Tuna silage generated comparable growth parameters with that of standard fish meal-based diet.	Yathavamoorthi et al. 2015
	Tuna sidestream solids	Tuna sidestream acid silage	Broiler chicken	4% inclusion of tuna sidestream silage indicated positive response in boilers regarding body weight and meat protein conversion.	Widjastuti et al. 2011
	Tuna sidestream solids	Tuna sidestream acid silage	Pacific white shrimp (*Litopenaeus vannamei*)	25% inclusion of tuna sidestream silage along with 75% of soyabean meal generated comparable growth results with that of fish meal control.	Hernández and Olvera-Novoa 2017
	Tuna sidestream solids	Tuna sidestream hydrolysate	Asian seabass/barramundi (*Lates calcarifer*)	The simultaneous subjunction of tuna sidestream hydrolysate and insect larvae significantly improved the growth, meat quality, gut health, and immune traits of juveniles in comparison to fish meal.	Chaklader et al. 2021
	Tuna sidestream solids	Tuna sidestream hydrolysate/meal	Nile tilapia (*Oreochromis niloticus*)	The combination at 20% of tuna sidestream meal and blood meal significantly increased the weight gain and specific growth ratio.	Mokolensang et al. 2021
	Tuna sidestream solids	Tuna sidestream meal	Spotted red snapper (*Lutjanus guttatus*)	Tuna by-product meal could replace fish meal up to a level of 25–30% with comparable results.	Hernández et al. 2014
	Tuna sidestream solids	Tuna sidestream meal	Korean rockfish (*Sebastes schlegelii*)	Tuna by-product meal could replace fish meal up to a level of 30–40% with comparable results.	Jeon et al. 2014
	Tuna sidestream solids	Tuna sidestream meal	Abalone (*Haliotis discus*)	Tuna by-product meal could replace fish meal up to a level of 75% with comparable results.	Jung et al. 2016
	Tuna sidestream solids	Fermented tuna sidestream meal	Olive flounder (*Paralichthys olivaceus*)	Fermented tuna by-product meal could replace fish meal up to a level of 10–12% with comparable results.	Oncul et al. 2019
	Tuna sidestream solids	Tuna sidestream hydrolysate	Persian sturgeon (*Acipenser persicus*)	Tuna viscera protein hydrolysate up to a level of 10–25% enhanced larval performance.	Ovissipour et al. 2014

### Tuna protein hydrolysate as a source of bioactive peptides

The marine environment, compared to fresh and brackish water, provides certain conditions such as scarcity of light, significant salt concentrations, and water pressure that can create unique amino acid sequences (Li et al. 2020) with interesting and significant biological properties (Eghtedari et al. 2021). These bioactive peptide fragments generally consists of 2 to 20 amino acids are usually liberated from plant, microbial, or animal-based proteins through chemical, biological, or enzymatic degradation of protein molecules (Suo et al. 2022). In addition to the nutritional value, the bioactive peptides have important health significance owing to their significant biological properties such as antioxidant (Wen et al. 2020), anti-inflammatory (Chakrabarti and Wu 2015), antihypertensive (Zhu et al. 2022), hypolipidemic (Wang et al. 2020), anticancer, and immunoregulatory (Chalamaiah et al. 2018) functions. Tuna being a rich source of protein provides an opportunity for the industry to extract valuable bioactive peptides from different sidestream sources.

Kiettiolarn et al. (2022) standardized the hydrolysis parameters for harvesting antioxidant peptides from tuna cooking juice concentrate (TCJC). The ideal hydrolysis conditions of Alcalase quantity (2.2% w/v) and hydrolysis period (281 min) resulted in maximum DPPH scavenging activity of 66.5% or 0.98 μmol Trolox/mg protein. The vacuum-dried portions (<5 kDa and >10 kDa) reported elevated DPPH radical scavenging activity, ABTS radical scavenging activity, and ferric-reducing antioxidant capacity. Unnikrishnan et al. (2021) utilized tuna (*Thunnus albacares*) dark meat, a by-product available from the canning industry as a source of antioxidant peptides. Enzymatic hydrolysis using papain with enzyme to substrate concentration of 0.98% E/S (w/w), hydrolysis time 240 min at 60°C, and pH 6.5 was found to be the ideal conditions. Wang et al. (2022b) prepared protein hydrolysate from the milt of skipjack tuna (*Katsuwonus pelamis*) utilizing different proteolytic enzymes after defattening and a microwave pre-treatment. Hydrolysate prepared using Neutrase with a degree of hydrolysis of 29.5% had antioxidant properties, and approximately 13 oligopeptides with antioxidant activity were identified. Cai et al. (2022a) extracted myeloperoxidase enzyme activity inhibiting antioxidative peptides from tuna protein utilizing enzymatic (Alcalase) hydrolysis followed by purification with ultrafiltration and Sephadex G-15 gel filtration. Fifty-three of the 55 peptide sequences identified showed myeloperoxidase enzyme activity inhibiting property. Zhang et al. (2019) utilized heads of skipjack tuna (*Katsuwonus pelamis*) from canning industry to isolate antioxidant peptides by employing enzymatic hydrolysis (pepsin and trypsin). Around 6 antioxidant peptides were isolated and purified using ultrafiltration and chromatography separation techniques. Peptides with high antioxidant activity were isolated from the cardiac arterial bulbs of skipjack tuna (*Katsuwonus pelamis*) by Cai et al. (2022b). Pepsin-assisted enzymatic hydrolysis conditions were standardized for the purpose. Utilizing ultrafiltration and chromatography, around eleven antioxidant peptide components were isolated, including four tripeptides and seven pentapeptides which showed elevated radical scavenging activities, protection against oxidative stress. Roe from skipjack tuna (*Katsuwonus pelamis*) were utilized by Wang et al. (2022a), to produce antioxidant peptides. The roes were defatted and subjected to microwave pre-treatment and then subjected to hydrolysis with 5 protease enzymes. The tuna roe protein hydrolysate generated using Flavourzyme reported maximum DPPH radical scavenging activity. Around 12 antioxidant peptides have been further isolated using ultrafiltration and electrophoretic methods. Some of the peptide fractions exhibited significant inhibition to lipid peroxidation, ferric reduction capacity, and protection of changes in Chang liver cells induced by H_2_O_2_.

### Recovery of collagen and gelatin from tuna skin

Gelatin is a hydrolytically degraded form of collagen, which is obtained from different animal components such as skin, connective tissue, tendons, and bones. There are 27 different varieties of this structural protein among which type I is the most common (Jiang 2015). Collagen has limited solubility which could be overcome by controlled denaturation through hydrolysis, transforming it into a functional, water-soluble polymer component called gelatin with wide application in the pharmaceutical, cosmetic, biomedical, and food industry (Kumar et al. 2017). The demand for this versatile biocomponent has been on the rise in recent times achieving a global production of 0.45 million tons in 2018 with an economical contribution of around 4.52 billion USD (Tkaczewska et al. 2018). Typically, 29–23% of the gelatin produced are from bovine skin and bones, 46% from pig skin, and 1.5% from fish (Sultana et al. 2018). This indicates the potential of seafood sidestreams to be utilized as a bovine or porcine alternative.

The concentration of imino acids such as proline and hydroxyproline which makes up to 12% of the collagen can be considered as an indicator of collagen content in that particular tissue (Risteli and Risteli 2006). The seafood gelatin sourced from fishes like tuna was observed to have an imino acids content of 17–20% (Shyni et al. 2014) while the mammalian gelatins were observed to have 30% (Poppe 1992). Glycine extracted from tuna is reported to constitute up to 32% of glycine of the total amino acid content along with a substantial proportion of hydroxyproline, proline, and alanine (Venugopal 2021). Tuna gelatin was also reported to have a viscosity 4.37cP and a bloom value of 177 (Shyni et al. 2014), while the commercial bovine collagen reported a viscosity of 13cP (Johnston-Banks 1990) and a bloom value up to 300 (Karim Rajeev and Bhat 2009). To overcome such shortcomings, attempts are being made to improve the gelation parameters of fish gelatin by incorporating polysaccharides from natural sources such as gellan and sodium alginate (Huang et al. 2019; Sow et al. 2019). Because of the religious concerns associated with mammalian gelatin, there is a trend to find alternative sources. Seafood processing sidestreams could be utilized as a good source for extracting gelatin to it (Hermida-Merino et al. 2022). Tuna as one of the largest marine pelagic seafood categories produces considerable quantities of processing sidestreams such as skin and bone, which qualifies it as a potential source for the extraction of fish gelatin.

#### Tuna collagen

Several methods have been used to isolate/extract collagen from tuna sidestreams. Ahmed et al. (2018) explored the possibilities of replacing pepsin derived from porcine sources with bacterial collagenolytic proteases for collagen extraction. The total collagen yield was reported to be from 17 to 18% for both the strains with FTIR and SDS-PAGE analysis confirming their type I characteristics. Kaewdang et al. (2014) extracted acid and enzyme (pepsin) soluble collagen from yellowfin tuna (*Thunnus albacares*) swim bladders. The yield was around 1.07% for collagen extracted using the acid method and 12.10% for collagen extracted using pepsin. The electrophoretic analysis proved that the extracted collagens were of type I. Ahmed et al. (2019) extracted collagen from the skin, scale, and bone of big eye tuna (*Thunnus obesus*) using both acid and enzymatic (pepsin) methodologies with corresponding yields of 13.5% and 16.7% from skin, 4.6% (pepsin) from scale, and 2.6% (pepsin) from bones, respectively. They reported that all the extracted collagens were of type I with elevated thermal transition (31.6–33.7 °C) and denaturation values (31.1–32.2 °C). A method for extracting acid soluble collagen from the skin of albacore tuna (*Thunnus alalunga*) was studied by Hema et al. (2013). The extracted collagen was of type I with glycine (33%) being the dominant amino acid with high values of hydroxyproline (8%) and a yield of 13.97% (w/v of tuna skin).

#### Tuna gelatin

Montero and Acosta (2020) optimized the acid-based extraction conditions of yellowfin tuna (*Thunnus albacares*) skin gelatin. The acid concentration, treatment time, and treatment temperature were found to have a significant effect on gelatin yield and physical properties. The process was also successfully scaled up to a ratio of 80:1 (pilot scale:laboratory scale). Karayannakidis et al. (2014) analyzed the effect of acid concentration, pre-treatment time, extraction temperature, and extraction time on the yield, gel strength, viscosity, melting point, and sensory properties of gelatin extracted from the skin of yellowfin tuna (*Thunnus albacares*). The study showed that the ideal condition includes an initial alkaline pre-treatment in two stages with a following acid pre-treatment (0.1 mol/L acetic acid for 1 h) and later extracting at 55°C with water for 6 h. Membrane filtration was explored by Montero et al. (2022), in extracting tuna skin gelatin. The gelling and melting temperature of the tuna gelatin and the retentates was within a range of 10–13.4°C and 18–20.6°C, respectively. The gel strength values of the retentates from the different membranes were between 228 and 440 g as maximum force. Azhar et al. (2018) evaluated the effect of different alkaline and acidic pre-treatments on bigeye tuna skin. The study concluded that the experiment with 0.1% alkaline (NaOH) and acidic (H_2_SO_4_) treatment and a soaking period of 48 h with a 60 °C hot water extraction for 5 h was the ideal combination for the extraction of gelatin from tuna skin based on the physical and chemical properties analyzed. Table 6 depicts the highlights of the technologies adapted for extraction of type I collagen/gelatin from tuna sidestreams.

**Table 6:** Collagen/gelatin extraction from tuna sidestreams

Sl. no.	Tuna species	Sidestream source	Type of collagen	Extraction conditions and yield	References
1	Bigeye tuna (*Thunnus obesus*)	Skin	Type I	Collagenolytic proteases from *Bacillus cereus* (FORC005) and *Bacillus cereus* (FRCY9-2) Collagen yield: FORC005: 188 g/kg FRCY9-2: 177 g/kg	Ahmed et al. 2018
2	Albacore tuna (*Thunnus alalunga*)	Skin	Type I	0.5M acetic acid Collagen yield: 13.97%	Hema et al. 2013
3	Bigeye tuna (*Thunnus obesus*)	Skin	Type I	Alkaline/acid pre-treatment (0.1% NaOH and H_2_SO_4_) Soaking time: 48 h at hot water extraction: 60 °C for 5 h Gelatin yield: 19.67%	Azhar et al. 2018
4	Yellowfin tuna (*Thunnus albacares*)	*Swim bladders*	Type I	*Acid and pepsin hydrolysis* Pre-treatment: 0.15 M NaOH 1:10 (w/v) at 4°C Defattening: 10% butyl alcohol sample/solvent ratio of 1:10 (w/v) for 12 h *Acid* *Hydrolysis*: 0.5 M acetic acid with a sample to solvent ratio of 1:10 (w/v) for 48 h at 4°C. *Pepsin hydrolysis*: 0.5 M acetic acid containing crude stomach extract (20 units/g swim bladder) using a solid/solution ratio of 1:10 (w/v) for 48 h at 4°C Collagen yield: *Acid method: 1.07%* *Pepsin method: 12.10%*	Kaewdang et al. 2014
5	Skipjack tuna (*Katsuwonus pelamis*)	Spine and skull	Type I	*Acid and pepsin hydrolysis* Pre-treatment: 0.01 mol·L^−1^ NaOH at a solid to alkali solution ratio of 1:10 (W/V) for 6 h demineralized with 0.5 mol·L^−1^ EDTA-2Na (pH 7.4) (solid to solution ratio 1:10W/V) for 48 h 0.5 mol·L^−1^ acetic acid with a solid to solvent ratio of 1:15 (W/V) for 48 h Yield (spine): *Acid method:* 2.47% *Pepsin method:* 5.62% Gelatin yield (skull): *Acid method:* 3.57% *Pepsin method:* 6.71%	Yu et al. 2014
6	Big eye tuna (*Thunnus obesus*)	Skin, scale, and bone	Type I	*Acid and pepsin hydrolysis* Pre-treatment: 0.1 M NaOH with continuous stirring for 24 h, 40 volumes of 0.5 M acetic acid for 3 days treated with 0.5% pepsin of 250 units/mg dry matter (w/v) (0.2 g pepsin/g of substrate) in 40 volumes of 0.5 M acetic acid for 48 h Collagen yield (skin): *Acid method:* 13.5% *Pepsin method:* 16.7% Yield (scales): *Pepsin method:* 4.6% Yield (bones): *Pepsin method:* 2.6%	Ahmed et al. 2019
7	Yellowfin tuna (*Thunnus albacares*)	Skin	Type I	Alkaline pre-treatment in two stages with a following acid pre-treatment (0.1 mol/L acetic acid for 1 h) and later extracting at 55°C with water for 6 h Gelatin yield (skin): 11.02%	Karayannakidis et al. 2014

### Tuna sidestreams as a source of enzymes

Enzymes are an important biochemical component in major physiological functions of organisms including human beings. The digestive enzymes aid in the breakdown of nutritional components in the diet which are necessary for building and repairing body tissue as well as controlling major functions in the human body (Patil et al. 2022). The modern lifestyle along with aging has been reported to significantly affect the human digestive system (Patil et al. 2022). The secretion of proteolytic enzymes such as pepsin was reported to decrease by 40% in the geriatric population due to various health conditions (Lee et al. 2022) which further results in a reduced ability to absorb nutrients from the diet (Buamard et al. 2020). This indicates the significance of supplementing digestive enzymes for the affected populations through diet to mitigate the challenge. Popularly bovine and porcine-isolated pepsin is commercially used for this purpose (Jurado et al. 2012). Around 60% of the total enzyme market is made up by the protease enzymes with numerous applications in industries such as detergent, leather, food, paper, photography, and bioremediation (Naveed et al. 2021). These enzymes could also be applied in therapeutics for inflammation and lesions (Fazilat 2016). Religious and cultural taboos have accelerated the search for alternative sources for digestive enzymes which has further aroused the interest in seafood sidestreams as a source for digestive enzymes (Nalinanon et al. 2010). The tuna viscera which is discarded as sidestream during the pre-processing stages is rich in proteolytic pepsinogen enzymes due to its stringent predatory diet. This provides an opportunity to the commercial enzyme industry to explore the possibilities to utilize tuna sidestreams as alternative source for enzyme harvesting.

Crude pepsin with comparable levels of hydrolytic activity to commercial pepsin extract was prepared from tuna viscera by Patil et al. (2022). Pasaribu et al. (2018) extracted pepsin from tuna (*Thunnus albacares*) visceral wall fluid using Tris-HCl buffer and fractional precipitation with ammonium sulfate and dialysis. The extracted crude pepsin (30–40% fraction) had a specific activity of 4.274 U/mg. Trypsin-like serine proteinases isolated from albacore tuna (*Thunnus alalunga*) spleen were found to have ideal enzyme activity at pH 9.5 and 50°C (Poonsin et al. 2017). Proteases extracted from albacore tuna (*Thunnus alalunga*) liver were observed to be with in the molecular weight range of 21–34 kDa (Sripokar et al. 2016).

### Recovery options for oil and liquid sidestreams from tuna processing

Tuna processing methods such as canning involve stages like pre-cooking, to ensure the ideal textural, color, microbial, and moisture levels are maintained in the final product. These pre-processing methods generate considerable quantities of solid and liquid sidestreams, rich in semisolids and fat that can be an open opportunity for the industry to recover these valuable nutritional components (Venugopal and Sasidharan 2021). Tuna canning industry produces condensates and liquid components which are abundant in proteins and fat (Sanchart et al. 2018b). Tuna oil is reported to be a rich source of EPA (5.11%) and DHA (23.89%) with a total PUFA content of 43% (Su Xiurong et al. 2015). Different techniques are employed for extraction of lipid from seafood sidestreams throughout the industry among which wet reduction is the most common.

Nazir et al. (2017) utilized tuna heads for extracting lipids using wet reduction, acid ensilation, and solvent extraction. Wet rendering resulted in the highest yield (12.8%) followed by solvent extraction (8.49%) and acid silage (6.16%). Taati et al. (2018) extracted oil from tuna sidestreams using wet reduction and enzyme extraction. Both methods gave a similar yield for EPA and DHA (27.3% and 29.32%). Sidestreams from Atlantic bluefin tuna (*Thunnus thynnus*) were used by Cutajar et al. (2022) for the extraction of oil employing three different methods: cold extraction, warm extraction, and enzymatic (pepsin) extraction. The enzyme extraction method resulted in maximum yield (29%) compared to other methods with a high content of EPA and DHA. Ferdosh et al. (2016b) utilized supercritical fluid extraction with carbon dioxide for extraction of lipid components from long tail tuna (*Thunnus tonggol*) heads under standardized conditions. de Oliveira et al. (2017) extracted oil from the head of yellowfin tuna (*Thunnus albacares*) using physical reduction, solvent extraction, and enzymatic separation with a PUFA yield of 39% for enzymatically isolated tuna oil. Suriani and Komansilan (2019) utilized sidestream oil from tuna canning industry to enrich and separate omega-3 fatty acids using urea crystallization technique. The concentrated oil was reported to have a PUFA concentration of 73%. Messina et al. (2022) utilized urea complexation and short path distillation (SPD) to enrich the PUFA fraction obtained from tuna oil recovered from the filleting sidestreams of bluefin tuna (*Thunnus thynnus*) and obtained a PUFA content of 85%. SPD was also employed by Messina et al. (2021) to enrich the PUFA fraction of the fatty acid ethyl esters obtained from refined viscera oil of farmed gilthead sea bream (*Sparus aurata*) obtaining a concentration of PUFA of 56%. Transesterification was used by Rosyadi et al. (2020) to convert oil from the tuna canning industry sidestream into biodiesel. The biodiesel obtained reported fuel characteristics such as density (849 kg/m^3^), viscosity (3.53 cSt), moisture content (0.6%), calorific value (9390 cal/gr), flash point (84° C), and acid number (3.6343 mg KOH/gr), respectively.

Khongnakorn and Youravong (2016) isolated protein from tuna cooking juice using forward osmosis as a low-energy intensive process, which elevated the protein recovery concentration to the level of 9% with a standard permeate flux (2.54 L/m^2^h). Kanpairo et al. (2012) prepared spray dried tuna flavor powder from tuna pre-cooking juice from the canning industry. The pre-cooking juice was initially concentrated through centrifugation to achieve a total soluble solid level of 15%. The process produced a tuna flavor powder with a pale brown coloration having moisture percentage of 4.63–7.46%, protein content of 28.49–42.06%, and an ash content of 3.44–4.25%. The extracted tuna flavor powder finds potential application as a flavoring agent in human as well as pet food. Ahmed et al. (2017) compared supercritical carbon dioxide extraction (SE) method and traditional Soxhlet extraction method using hexane (HE) for isolating oil from tuna sidestreams such as skin, scales, and bones. The SE oil was superior to the HE with respect to lower heavy metal concentration, better color, viscosity, and lower acid, peroxide, and free fatty acid values. SE method was also utilized by Ferdosh et al. (2016a) to extract fish oil from tuna sidestreams with positive results reporting a PUFA yield of 48.93%. Šimat et al. (2020) qualitatively evaluated different tuna sidestreams such as tuna entrails and tuna liver for estimating the potential for fish oil extraction. The tuna entrails reported a total fish oil yield of 26.1% and tuna liver a total fish oil yield of 33.4%, emphasizing the oil extraction potential of the tuna sidestreams.

### Miscellaneous technologies adopted for tuna sidestream recovery

Apart from widely adopted technologies such as meal production and hydrolysate, gelatin, and enzyme extraction from tuna sidestreams, many other technologies have been introduced for isolating different nutritional components from the rest raw materials. Yoon et al. (2019) prepared protein isolates from yellowfin tuna (*Thunnus albacares*) roe using isoelectric solubilization precipitation (ISP) technique. The protein isolates from tuna roe were found to exhibit good buffering capacity, foaming ability, and emulsifying ability including antioxidative and antihypertensive activity. Sanchart et al. (2018a) utilized tuna condensate, a liquid sidestream available from the canning industry to isolate gamma-aminobutyric acid (GABA) via glutamic acid by converting glutamine, demonstrating the suitability of tuna canning sidestreams to be utilized as a source for extraction of GABA on an industrial scale. Sumogod et al. (2020) extracted hyaluronic acid (HA) from yellowfin tuna (*Thunnus albacares*) eyeballs through standardized process of tissue extraction, tissue hydrolysis through enzymatic method, cetylpyridinium chloride (CPC)-sodium chloride (NaCl)-induced precipitation, filtration, diafiltration, and alkaline hydroalcoholic precipitation followed by freeze drying. The ideal isolation conditions were observed to be recovery and fractionation concentration (3% CPC:3M NaCl) and supernatant:ethanol ratio (1:3 mL·mL^−1^) for alcoholic precipitation. Murthy et al. (2014) extracted bone powder from yellowfin tuna (*Thunnus albacares*) through alkaline deproteinization, drying, and pulverizing. The bone powder was analyzed for elemental composition and was found to be a rich source of calcium and phosphorus with a Ca:P ratio of 2.5–3.3:1. The powder was also found to be devoid of any heavy metal impurities such as cadmium and mercury. Pallela et al. (2011) isolated microstructured and nanostructured hydroxyapatite (HAp) from big eye tuna (*Thunnus obesus*) bone through thermal calcination technique in the presence of synthetic polymers. The study demonstrated that the hydroxyapatite produced in the presence of the polymers had better crystallinity and biocompatibility properties.

## Green extraction techniques for tuna sidestream recovery

Other than the conventional processes used for the recovery of tuna sidestreams, which usually employs strong chemicals, dangerous to the environment, expensive, and highly water consuming during the washing and neutralization stages, there are green technologies available to be employed for a more safe and sustainable recovery process (Venugopal and Sasidharan 2022). The green processes are generally less toxic with minimal environmental impact, with reusable option and highly efficient in extraction potential (Vicente et al. 2022). The sustainable technologies which come under the green category are enzymatic processes, methanogenesis, microbial fermentation, photosynthesis, oleaginous processes (Venugopal and Sasidharan 2022), ultrasound-assisted techniques, high hydrostatic pressure, microwave-assisted processes, pulsed electric field, dense phase carbon dioxide, membrane filtration, supercritical and subcritical fluids (Ali et al. 2021), and utilization of green solvents like water, ionic solvents, liquid polymers, acetone, methanol, ethanol, and propanol (Vicente et al. 2022). Saidi et al. (2014) hydrolyzed tuna dark muscle sidestream using Alcalase and applied a combined process of ultra and nanomembrane filtration in order to isolate peptides according to their molecular weight (1–4 kDa) with positive results. Yoon et al. (2019) successfully isolated fish roe protein from yellowfin tuna (*Thunnus albacares*) using alkaline solubilization and acid precipitation (pH 11/4.5). Isoelectric solubilization/precipitation (ISP) was successfully employed by Lee et al. (2016) for isolating tuna roe proteins from yellowfin tuna under several solubilization and precipitation conditions. The study observed that the isolated proteins maintained the functional and nutritional characteristics of the native protein. Cha et al. (2020) also utilized ISP method for isolating roe protein from skipjack tuna with similar results. de Oliveira et al. (2016) extracted oil from tuna sidestreams using enzymatic hydrolysis with Alcalase with an enzyme to sidestream ratio of 1:200 (w/w) at 60°C for 120 minutes and pH 6.5. Fang et al. (2019) compared different green technologies like supercritical carbon dioxide fluid extraction (SFE-CO2), subcritical dimethyl ether extraction (SDEE), and enzymatic extraction (EE) for extracting tuna oil rich in n-3 polyunsaturated fatty acids (PUFAs), vitamin D, and vitamin A from tuna liver sidestreams. The green technologies when compared to traditional extraction methods were successful in preventing the loss of valuable nutrients like vitamins as well as avoiding the deterioration of PUFA. Figure 3 shows the green technology options applicable to sidestream resource recovery from tuna.

## Future outlook

**Fig. 2:**
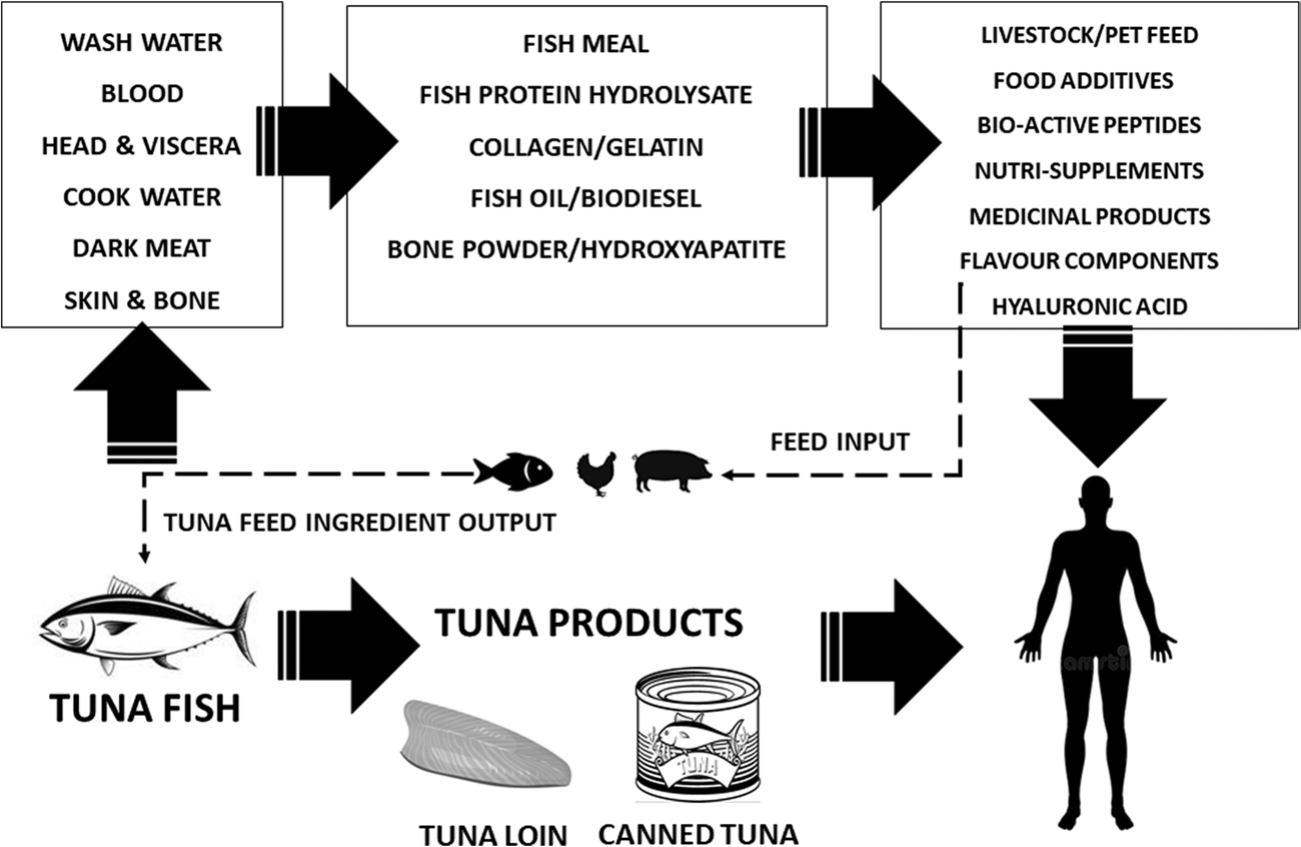
The circular blue economy perspective of tuna sidestream valorization

A seafood sidestream valorization system with a circular economic perspective is necessary to foster an ecosystem with environmental socioeconomic sustainability (Bhat 2021). Similarly, tuna sidestream biomass, which is an integral part of the blue bioeconomy, could be transformed into a circular economic framework, if proper utilization technologies are adopted. This is also important in the context of the Sustainable Development Goals (SDGs), proposed by the United Nations aiming to achieve an end to different disparities in the food delivery system such as poverty and hunger by the year 2030 (United Nations 2015). The SDG 14 and 12.3 are particularly important considering seafood sidestream management as they concentrate on sustainable development of the marine environment and reducing food sidestream and food losses including the seafood sidestreams (Duarte et al. 2020). For successful implementation of the sidestream utilization and management plan, the tuna processing industry also needs to address certain challenges in the near future. One such challenge is the availability of proper logistics and handling infrastructure to guarantee the quality of the sidestreams generated over the period of time to sustain a sidestream valorization industry (Shahidi et al. 2019; Shavandi et al. 2019; Coppola et al. 2021). Another roadblock which needs to be addressed is the availability of valorization technologies. These are mostly developed at a laboratory scale rather than industrial scale. This limits their industrial adaptation, and they need to be upscaled into a biorefinery concept in the most cost-effective manner possible (Mohan et al. 2020; Coppola et al. 2021). Other than those, an awareness among the stakeholders regarding the status of sidestreams as an equivalent raw material as of the primary produce needs to be generated (Guillen et al. 2018; Mo et al. 2018). Figure 2 represents the circular blue bioeconomical perspective of tuna sidestream valorization as a blueprint for policymakers and industrialists to align their tuna utilization cycle with sustainable goals put forward by the United Nations.

## Conclusion

**Fig. 3:**
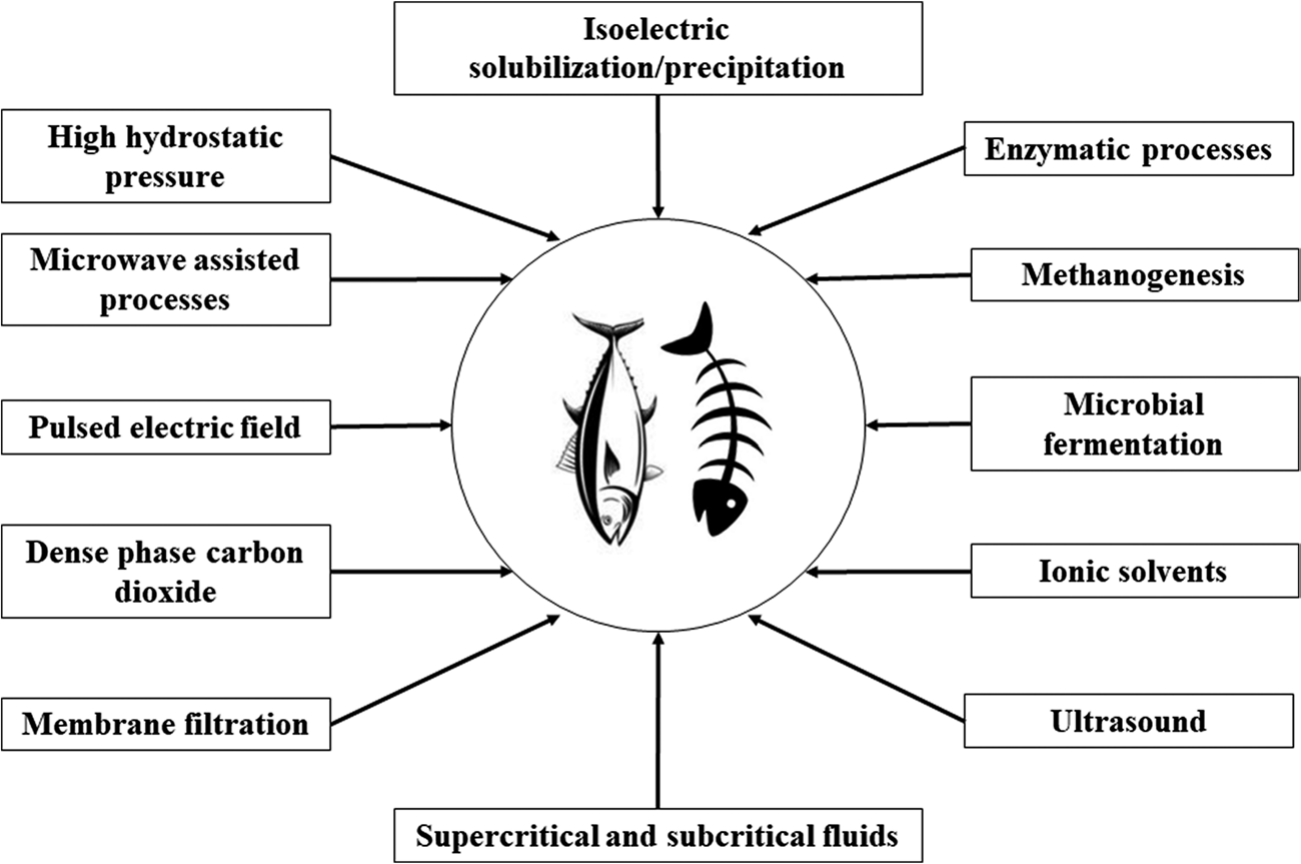
Green technologies for tuna sidestream valorization

The tuna fisheries in general are currently facing sustainability issues associated with the fishing methods as well as the huge quantity of sidestreams generated during the pre-processing and processing stages. The systematic utilization of solid and liquid sidestreams generated is essential to make sure the significant quantity of nutritional components associated with the sidestreams is available for the consumers in the form of value-added products. This article highlights the potential of tuna processing sidestreams as a valuable raw material for resource recovery. Protein, one of the prominent components of the tuna solid sidestreams, can be recovered as collagen, gelatin, hydrolysate, meal, and bioactive peptides with numerous applications in food, packaging, pharmaceutical, and animal nutrition industry. The fat from the sidestreams can be recovered as PUFA or converted into biofuel. The liquid sidestreams from tuna processing also hold potential for recovery of lipid and flavor components. The identification of these potential technologies along with their advantages and disadvantages can help the industry to adopt the optimum process according to the type and quantity of their sidestreams. Complementation of such technologies into the production process can ultimately result in more economically circular and sustainable tuna fisheries (Fig. 3).

## Data Availability

Not applicable.
